# Fatty corner lesions in T1-weighted magnetic resonance imaging as an alternative to sacroiliitis for diagnosis of axial spondyloarthritis

**DOI:** 10.1186/s41927-019-0068-5

**Published:** 2019-05-30

**Authors:** Ho Yin Chung, Rachel Sze Wan Yiu, Shirley Chiu Wai Chan, Kam Ho Lee, Chak Sing Lau

**Affiliations:** 1 Division of Rheumatology and Clinical Immunology, Department of Medicine, the University of Hong Kong, Pokfulam, Hong Kong, China; 2 Department of Radiology, Queen Mary Hospital, Hong Kong, China

**Keywords:** Spondyloarthropathies, Magnetic resonance imaging, Diagnosis, Back pain, Spine

## Abstract

**Background:**

A fatty corner lesion (FCL) is a well-demarcated fat infiltration in the corner of a vertebral body on T1 magnetic resonance imaging (MRI) sequence. It has been reported to be useful in the diagnosis of axial spondyloarthritis (axSpA). Our objective is to systematically evaluate the diagnostic accuracy of FCLs in tertiary centre patients with chronic back pain.

**Method:**

Two hundred and thirty eight axSpA patients and 62 non-axSpA patients with back pain were recruited from five rheumatology centres. Clinical, biochemical, and radiological parameters were collected and all patients underwent a MRI of the spine and sacroiliac (SI) joints. FCLs in vertebral bodies from C4 to L5 were scored. The number and location of FCLs were clustered together to determine an optimal combination for diagnosis. Results were compared with expert diagnosis as the “gold standard”.

**Results:**

FCLs of the anterior whole spine (AUC 0.62; *p* = 0.003) and anterior thoracic spine (AUC 0.64; *p* = 0.001) had diagnostic significance. Incorporating at least 5 whole spine FCLs into the imaging criteria of the Assessment of SpondyloArthritis international Society (ASAS) criteria for axSpA yielded a sensitivity of 91.6% and specificity of 91.9%. Similarly, applying at least 3 anterior thoracic FCLs to the imaging criteria of the ASAS axial SpA criteria yielded a sensitivity of 92.0% and specificity of 93.5%.

**Conclusion:**

FCLs could be used to diagnose axial SpA. The presence of at least 3 anterior thoracic FCLs in T1-weighted MRI spine suggests a diagnosis of axial SpA without additional MRI of the SI joints.

**Trial registration:**

The cohort has been registered in the clinical trial registry of the University of Hong Kong (HKUCTR-2087).

**Electronic supplementary material:**

The online version of this article (10.1186/s41927-019-0068-5) contains supplementary material, which is available to authorized users.

## Background

The Assessment of SpondyloArthritis international Society (ASAS) classification criteria for axial spondyloarthritis (axSpA) in 2009 [1, 2] was the first to incorporate sacroiliitis on magnetic resonance imaging (MRI) in its imaging criteria, which resulted in early diagnosis [3]. In addition to inflammation, MRI has been used to identify other lesions in axSpA. The “MR corner sign”, which includes both inflammatory and non-inflammatory lesions of the spine, was found to be specific for ankylosing spondylitis [4] prior to the introduction of the ASAS criteria. Fatty corner lesions (FCLs) in the lumbar spine were initially suggested in diagnosis of axSpA, and Bennett et al. had proposed the use of whole spine FCLs [5].

FCLs have not been included in the ASAS classification criteria due inconclusive evidence of diagnostic utility. In 2015, Weber et al. found that when used alone, whole spine FCLs showed poor positive and negative likelihood ratios (LR) in 130 patients with axSpA (for ≥6 FCLs, positive LR were 2.49–2.53; negative LR were 0.64–0.68) [6]. However, the SpondyloArthritis Caught Early (SPACE) cohort of 287 patients with chronic back pain in Europe found promising data for the diagnostic utility of FCLs. The presence of at least 5 fatty lesions on MRI spine had the ability to identify axSpA with a specificity of 95%. The proportion of patients with ASAS imaging arm criteria positive axSpA with at least 5 fatty lesions ranged from 18.2 to 21.6%. [7]. In view of apparently conflicting findings, this study aims to systematically evaluate the diagnostic utility of FCLs at different vertebral levels in a large tertiary cohort of patients with chronic back pain.

## Methods

This is a multicentre retrospective study of prospectively acquired data. The acquired data was from a cohort originally used to evaluate the utility of diffusion weighted imaging (DWI) in the diagnosis and clinical monitoring of axSpA. The cohort has been registered in the clinical trial registry of the University of Hong Kong (HKUCTR-2087). Detailed methods have been described in our previous publication [8]. Recruitment started in May 2016 and is on-going. Data analysis was performed on results up to Feb 2018.

### Patient recruitment

Two groups of patients were recruited consecutively from five different rheumatology centres (Queen Mary Hospital, Tung Wah Hospital, Grantham Hospital, Pamela Youde Nethersole Eastern Hospital, and Tseung Kwan O Hospital) in Hong Kong. The first group consisted of 238 patients with a known diagnosis of axSpA and chronic back pain. This group was termed the “axSpA group”. The second group was the control group. It consisted of 62 patients with a known diagnosis of diseases other than SpA. The group included 43 patients with known spinal degeneration, 1 patient with fibromyalgia, 1 patient with tuberculosis of spine and 17 patients with non-specific back pain and was termed the “non-axSpA group”. Written informed consent was obtained from all subjects. Exclusion criteria included: i) pregnancy, ii) inability or refusal to undergo MRI examination, iii) inability to give written consent.

### Clinical and demographic data

Clinical and demographic data were collected from recruited patients. These included: age, sex, history of smoking and regular use of alcohol, duration and severity of back pain (scored on a 1-to-10 numeric rating scale), and family history of SpA. Physical examination was performed for tender/ swollen joint counts, Maastrich Ankylosing Spondylitis Enthesitis Score (MASES) [9], Bath Ankylosing Spondylitis Metrology Index (BASMI) [10], and number of dactylitis sites. All patients completed three questionnaires: Bath Ankylosing Spondylitis Disease Activity Index (BASDAI) [11], Bath Ankylosing Spondylitis Functional Index (BASFI) [12], and Bath Ankylosing Spondylitis Global Index (BASGI) [13]. Blood parameters including human leucocyte antigen (HLA) B27, C-reactive protein (CRP) and erythrocyte sedimentation rate (ESR) were recorded. Ankylosing Spondylitis Disease Activity Index (ASDAS) were calculated based on both ESR (ASDAS-ESR) and CRP (ASDAS-CRP) [14, 15].

### Radiographic grading of lumbosacral (LS) spine and sacroiliac (SI) joints

Anteroposterior (AP) views of LS spine radiographs were done in all patients. SI joint radiographs were graded according to the Modified New York (MNY) criteria [16]: 0, normal; 1 suspicious; 2, sclerosis/ erosion with normal joint space; 3, sclerosis/ erosion with change in joint space or partial ankylosis; 4, complete fusion. Bilateral sacroiliitis grade 2 or above, or unilateral sacroiliitis grade 3 or above was defined as radiological AS. The gradings were only used for describing the percentage of radiological AS and not involved in the analyses.

### MRI scoring of the spine and SI joints

All recruited patients underwent whole spine and bilateral SI joint MRI examinations using a 3 T Achieva scanner (Philips Healthcare, Best, the Netherlands). A torso coil was used to image both the spine and sacroiliac joints. Short tau inversion recovery (STIR) sequence, T1 weighted, and DWI were obtained consecutively in the same MRI examination. Technical parameters for STIR images are summarized in Table 1. The imaging parameters and others (including DWI) have been reported in our previous publication [8].

**Table 1:** Imaging parameters for STIR, T1, and DWI sequences

	STIR	T1	DWI
TR/TE (ms)	5000/80	800/8	4000/90
Field-of-view (mm^2^)	150/240	150 × 240	300/241
Matrix size	152 × 157	168 × 217	124 × 100
Slice thickness (mm)	3.5	3.5	4
SENSE factor	N/A	N/A	2

*TR* repetition time, *TE* echo time, *SENSE* sensitivity encoding, *STIR* short tau inversion recovery, *DWI* diffusion weighted imaging, *N/A* not applicable

**Fig. 1:**
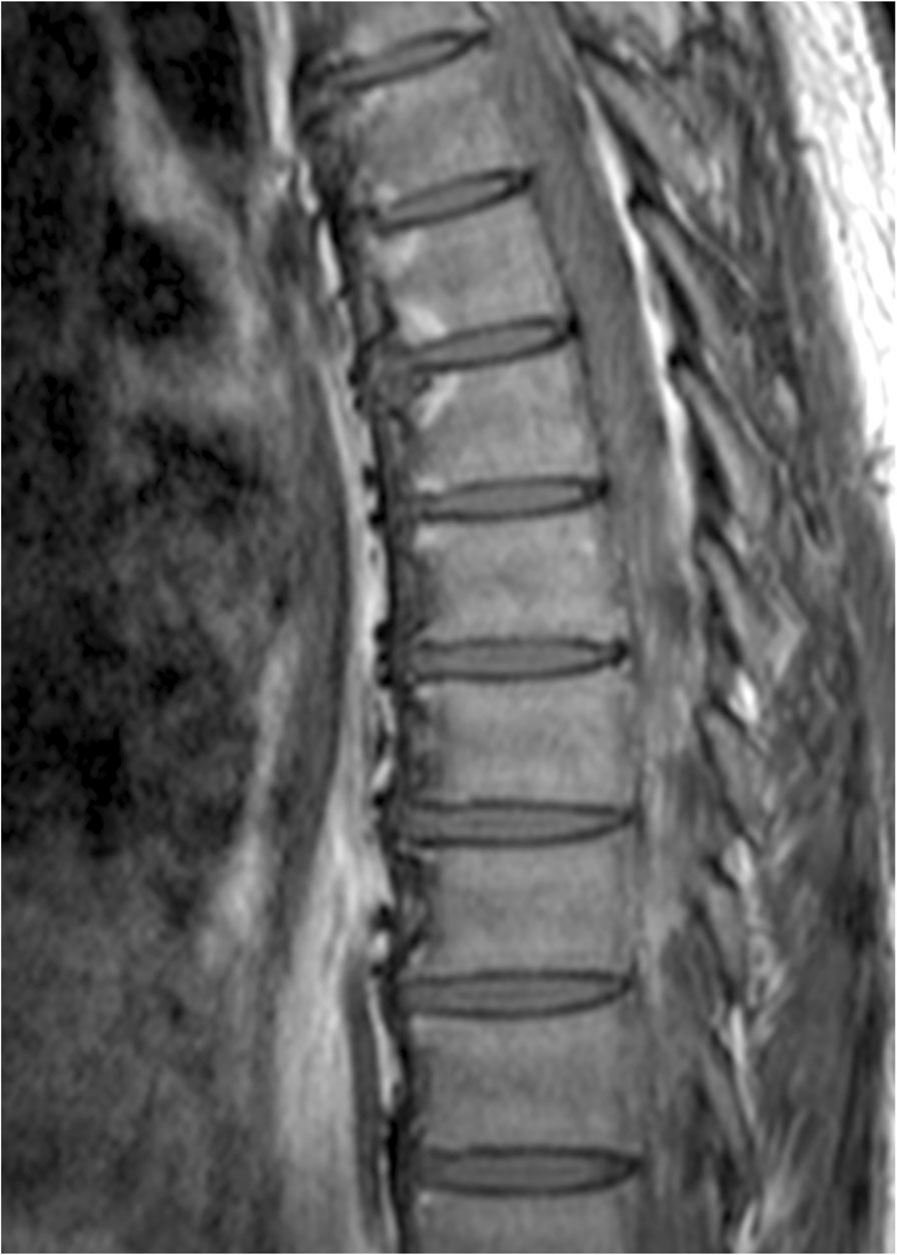
Fatty corner lesions

MRI of SI joints were scored according to the Spondyloarthritis Research Consortium of Canada (SPARCC) scoring system [17] by two independent readers (HYC, SCWC) blinded to clinical, radiological parameters and T1-weighted MRI spine. Positive sacroiliitis in STIR images was defined as: one signal of subchondral bone marrow edema on at least 2 slides, or more than one signal on a single slice, according to the ASAS handbook [18]. True sacroiliitis required identification by both readers. Similarly, FCLs in STIR and T1-weighted MRI spine were read by two independent readers (HYC, RSWY), blinded to clinical, radiological parameters and STIR images of SI joints. An FCL was defined as a well-demarcated triangular lesion, hyper-intense on T1-weighted images and suppressed on STIR sequences, present on at least one sagittal slice in the corner of any vertebral body as previously described by Bennett et al. (Fig. 1) [5]. As the upper vertebrae have different anatomical shapes and could be difficult to score, our scorings involved only C4 to L5 vertebral bodies. Both anterior and posterior vertebral corners were scored.

### Statistical analyses

Student’s t-tests and chi square tests were used to compare baseline demographic and clinical characteristics in the axSpA and non-axSpA groups. The Student’s t-test was also used to compare the number of FCLs in the two groups. At the vertebral levels in which significant differences were found, receiver operating characteristic (ROC) curves were constructed. The area under the curve (AUC) was used to determine the optimal cut off number of FCLs for diagnostic utility. This was done by selecting the number with the highest sensitivity corresponding to a specificity (95.2% as stated in the results section) greater than that of the imaging arm of the ASAS axSpA criteria in order not to jeopardize the specificity of the new criteria.

Upon 1) replacing the sacroiliitis on imaging criteria in ASAS criteria with the optimal cut off number of FCLs and 2) including the optimal cut off number of FCLs into the ASAS criteria. We determined their sensitivity, specificity, positive predictive value (PPV), negative predictive value (NPV), positive likelihood ratio (LR+) and negative likelihood ratio (LR-). Effect of LR+ was defined as none, slight increase, moderate increase, large increase for the groups with values 1–2, 2–5, 5–10, and > 10 respectively. Similarly, effect of LR- was defined as none, slight decrease, moderate decrease, large decrease for the groups with values 1–0.5, 0.5–0.2, 0.2–0.1, and < 0.1 respectively.

Cohen’s kappa was used to measure agreement in MRI scoring (FCLs and sacroiliitis) by the two readers. Value ranges of 0.00–0.20, 0.21–0.40, 0.41–0.60, 0.61–0.80, 0.81–1.00 represented slight, fair, moderate, substantial, and near perfect agreement respectively [19].

## Results

Clinical history, physical examination and laboratory findings of the axSpA group and non-axSpA group are described in Table 2. When compared to the non-axSpA group, axSpA patients had longer duration of back pain (12.0 ± 11.4 vs 7.1 ± 8.4; *p* = 0.002), higher levels of CRP (1.07 ± 1.94 vs 0.37 ± 1.27; *p* = 0.001), male predominance (55.0% vs 26.2%; *p* < 0.001), and greater proportion with HLA-B27 positivity (76.8% vs 16.0%; *p* < 0.001).

**Table 2:** Clinical history, physical examination and laboratory findings of the studied patients

	axSpA (*n* = 238) Mean ± SD/ (percentage)	Non-axSpA (*n* = 62) Mean ± SD/ (percentage)	*p*-value
Age (years)	43.5 ± 13.1	47.1 ± 17.1	0.13
Duration of back pain (years)	12.0 ± 11.4	7.1 ± 8.4	0.002
Male sex	131 (55.0%)	16 (26.2%)	< 0.001
HLA-B27 positivity	169 (76.8%)	8 (16.0%)	< 0.001
Smoker	66 (27.8%)	11 (19.0%)	0.17
Regular alcohol use	27 (11.6%)	5 (5.3%)	0.16
Radiologic AS	149 (62.9%)	0 (0.0%)	< 0.001
Back pain NRS	5.74 ± 2.35	6.10 ± 2.27	0.32
BASDAI	4.82 ± 2.00	NA	–
BASFI	3.12 ± 2.44	NA	–
Tender joint count	1.57 ± 3.02	2.74 ± 4.66	0.07
Swollen joint count	0.60 ± 1.57	0.97 ± 2.58	0.31
Enthesitis score	0.43 ± 0.90	0.58 ± 1.80	0.53
Number of dactylitis	0.19 ± 1.02	0.26 ± 1.47	0.66
BASMI	3.44 ± 1.65	NA	–
ESR (mm/hr)	32.9 ± 25.6	34.5 ± 26.2	0.68
CRP (mg/L)	1.07 ± 1.94	0.37 ± 1.27	0.001
Presence of FCL at C-spine	28 (11.8%)	2 (3.2%)	0.05
Presence of FCL at T-spine	91 (38.2%)	8 (12.9%)	< 0.001
Presence of FCL at L-spine	85 (35.7%)	16 (25.8%)	0.14
BASGI	5.38 ± 2.49	NA	–
ASDAS ESR	3.03 ± 1.02	NA	–
ASDAS CRP	1.91 ± 0.83	NA	–

*HLA* Human Leucocyte Antigen, *ASAS* Assessment of SpondyloArthritis international Society, *IBP* inflammatory back pain, *axSpA* axial spondyloarthritis, *AS* ankylosing spondylitis, *NRS* numerical rating scale, *BASDAI* Bath Ankylosing Spondylitis Disease Activity Index, *BASFI* Bath Ankylosing Spondylitis Functional Index, *BASMI* Bath Ankylosing Spondylitis Metrology Index, *ESR* erythrocyte sedimentation rate, *CRP* C-reactive protein, *BASGI* Bath Ankylosing Spondylitis Global Index, *ASDAS* Ankylosing Spondylitis Disease Activity Index, *FCL* fatty corner lesion, *C-spine* cervical spine, *T-spine* thoracic spine, *L-spine* lumbar spine, *NA* not applicable

**Fig. 2:**
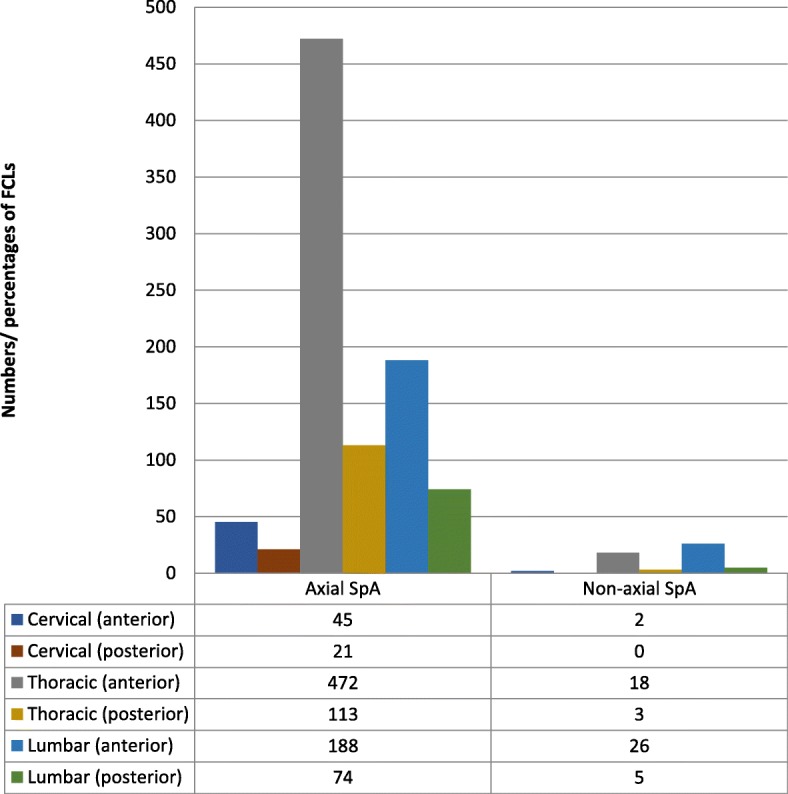
Distribution of FCLs in axial SpA and non-axial SpA groups

The agreement in identifying FCLs between the two readers (HYC, RSWY) was substantial (Cohen’s kappa 0.65 ± 0.03). In the axSpA group, FCLs were preferentially distributed at the thoracic level (585/913 or 64.1%), in contrast to distribution at the lumbar level (31/54 or 57.4%) in the non-axSpA group (Fig. 2). Overall, more FCLs were found in the axSpA group than the non-axSpA group (3.8 ± 6.8 vs 0.9 ± 2.2; *p* < 0.001) (Table 3).

**Table 3:** Number of FCLs in axial SpA and non-axial SpA groups

	Mean FCLs in axSpA group ±SD	Mean FCLs in non-axSpA group±SD	*p*-value
All spinal lesions	3.8 ± 6.8	0.9 ± 2.2	< 0.001
Anterior lesions	3.0 ± 5.1	0.8 ± 1.9	< 0.001
Posterior lesions	0.9 ± 2.4	0.1 ± 0.5	< 0.001

*FCL* fatty corner lesion, *axSpA* axial spondyloarthritis, *SD* standard deviation

ROC curves were used to determine the diagnostic utility of FCLs at different vertebral levels (Table 4). Whole spine FCLs (AUC 0.615, 95% CI 0.545; 0.685, *p*-value 0.01), thoracic spine FCLs (AUC 0.638, 95% CI 0.570; 0.707, *p*-value 0.001), anterior whole spine FCLs (AUC 0.622, 95% CI 0.551; 0.692, *p*-value 0.003), and anterior thoracic FCLs (AUC 0.640, 95% CI 0.572; 0.708, *p*-value 0.001) all had the ability to differentiate between patients with and without axSpA. At the lumbar spinal level, anterior FCLs showed only a tendency to be able to differentiate between patients with and without axSpA (AUC 0.576, 95% CI 0.500; 0.652, *p*-value 0.07).

**Table 4:** Diagnostic utility and cut-off values of FCLs at different spinal levels in diagnosing axSpA

	AUC	Stand Error	*p*-value	95% CI
All spinal lesions				
Whole spine	0.615	0.036	0.01	0.545; 0.685
Cervical	0.544	0.039	0.29	0.467; 0.620
Thoracic	0.638	0.035	0.001	0.570; 0.707
Lumbar	0.561	0.039	0.14	0.486; 0.637
Anterior lesions				
Whole spine	0.622	0.036	0.003	0.551; 0.692
Cervical	0.535	0.040	0.40	0.457; 0.612
Thoracic	0.640	0.035	0.001	0.572; 0.708
Lumbar	0.576	0.039	0.07	0.500; 0.652
Posterior lesions				
Whole spine	0.567	0.038	0.11	0.492; 0.641
Cervical	0.521	0.040	0.61	0.442; 0.600
Thoracic	0.560	0.038	0.15	0.485; 0.635
Lumbar	0.527	0.040	0.52	0.449; 0.605
		Cut-off threshold (no of FCLs)	Sensitivity (%)	Specificity (%)
Anterior whole spine FCLs		≥3	26.9	93.5
		≥4	22.7	95.2
		≥5	20.6	96.8
Anterior thoracic FCLs		≥1	31.5	90.3
		≥2	26.5	95.2
		≥3	18.5	98.4

*FCL* fatty corner lesion, *axSpA* axial spondyloarthritis, *AUC* area under curve, *CI* confidence interval

Agreement between the two readers in reading sacroiliitis on MRI according to the ASAS criteria was substantial (Cohen’s kappa 0.79 ± 0.04). From our analyses, posterior FCLs had no diagnostic utility. The diagnostic value of whole spine FCLs was predominantly contributed by that from anterior FCLs (Table 3), therefore only anterior FCLs were used to determine optimal cut-off values. The optimal number of FCLs corresponded with a specificity greater than that of the ASAS criteria. The ASAS criteria had a sensitivity of 90.3% and specificity of 93.5% while its imaging arm had a sensitivity of 84.5% and specificity of 95.2%. The different cut-off thresholds of FCLs are presented in the lower part of Table 4. With a specificity greater than 95.2%, the optimal cut-off values for anterior whole spine FCLs was ≥5, and anterior thoracic FCLs was ≥3. Different scenarios of the ASAS criteria in diagnosing axSpA are presented in Table 5. The greatest sensitivity was obtained by including ≥3 anterior thoracic FCLs to the imaging arm of the ASAS axSpA criteria.

**Table 5:** Sensitivity, specificity, positive predictive value, negative predictive value, positive and negative likelihood ratio in different scenarios of the ASAS axSpA criteria in the diagnosis of axSpA

	Sensitivity (%)	Specificity (%)	PPV (%)	NPV (%)	LR+	LR-
ASAS axSpA criteria (unaltered)	90.3	93.5	90.3	93.5	13.89	0.10
Imaging arm only	84.5	95.2	89.5	95.2	17.60	0.16
Sacroiliitis replaced by ≥3 anterior thoracic FCLs	80.6	93.4	98.0	55.3	12.2	0.21
Sacroiliitis replaced by ≥5 anterior whole spine FCLs	79.4	93.4	97.9	53.8	12.0	0.22
Sacroiliitis or ≥ 3 anterior thoracic FCLs	92.0	93.5	92.0	93.5	14.15	0.09
Sacroiliitis or ≥ 5 anterior whole spine FCLs	91.6	91.9	91.6	91.9	11.31	0.09

*PPV* positive predictive value, *NPV* negative predictive value, *LR* + positive likelihood ratio, *LR-* negative likelihood ratio, *ASAS* Assessment of SpondyloArthritis international Society, *axSpA* axial spondyloarthritis, *FCL* fatty corner lesion

### Presence of FCLs without sacroiliitis

Thirteen patients (out of 235 patients or 5.5%) with expert diagnosis of SpA, had ≥3 anterior thoracic FCLs but without sacroiliitis on both x-ray and MRI. Similarly, 12 patients (out of 235 patients or 5.1%) with expert diagnosis of SpA, had ≥5 whole spine FCLs but without sacroiliitis on both x-ray and MRI.

## Discussion

We evaluated and reported the diagnostic utility of anterior FCLs on T1 MRI in axSpA. In patients with features of SpA, replacing sacroiliitis on imaging with FCLs had only fair sensitivity but good specificity. Aadding FCLs into the imaging entry criteria only slightly improved the sensitivity.

Acute inflammatory lesions in the vertebrae affects the thoracic spine preferentially over the cervical or lumbar spine [20, 21]. Baraliakos et al. [21] found definite thoracic spinal inflammation in 74% of AS patients, predominantly at the T7-T8 vertebral unit. As fatty changes are thought to be the result of inflammation [22], it may be deduced that FCLs would also occur predominantly in the thoracic spine. In fact, Kim et al. had reported FCLs to be more common in the anterior part of the lower thoracolumbar spine [4]. Bennett et al. also reported FCLs to be more common in the thoracic spine [5]. Our results are consistent with their reports.

FCLs at different vertebral levels have variable diagnostic utility. Kim et al. proposed using lumbar FCLs for diagnosis of AS [4]. However, our data showed only a tendency for lumbar FCLs to aid diagnosis. Weber et al. showed that whole spine FCLs ≥6 has poor likelihood ratios [6] as a diagnostic criteria. Its specificity of 81–82% was significantly less than that found in our study. In contrast, our data was more compatible with Kim et al. [4], and subsequently the SPACE cohort [7], in which whole spine FCLs ≥5 had a specificity of ≥95% in discriminating between axSpA and non-axSpA. Overall, lumbar and whole spine FCLs show variable diagnostic utility. Although FCLs are observed in axSpA, they are also present in degenerative and other spinal disorders [23] which could contribute to the differences in specificity. Among all vertebral levels, the thoracic spine is the least affected by degeneration [24, 25], rendering a more accurate diagnostic tool for axSpA.

The differential utility between anterior and posterior FCLs in diagnosis of axSpA is a new finding in our study. Anterior FCLs contributed predominantly to the overall diagnostic utility in whole spine FCLs because they were present much more frequently than posterior FCLs. Although both whole spine FCLs and anterior spine FCLs were useful in axSpA diagnosis, the former group was not included in analyses because of its lower AUC. Posterior FCLs were not useful owing to its low frequency of occurrence.

Unlike Weber et al. and the SPACE cohort, we did not use the Canada-Denmark scoring system [26, 27] because we wanted to differentiate the usefulness of FCLs at individual vertebral levels and bisected into anterior and posterior spine. In contrast, the Canada-Denmark scoring system gives a summative score of the whole spine. C2, C3 and S1 vertebral bodies were not evaluated in this study. Using our individual scoring, we also reported an optimum cut-off of ≥5 anterior whole spine FCLs, which is similar to previous studies [7].

Of all the combinations of FCLs evaluated, we found that at least 3 anterior FCLs at the thoracic level had the greatest diagnostic utility in axSpA. However, routine use of T1 MRI for axSpA diagnosis is not recommended because replacing the sacroiliitis criteria with FCLs or including them into the ASAS axSpA criteria have not shown to improve diagnostic sensitivity. There were only a few patients with significant number of FCLs but no sacroiliitis on x-ray or MRI. The high false positive values limit the sole use of FCLs in diagnosis of axSpA. However, in patients with clinical features of axSpA and incidental findings of at least 3 anterior thoracic FCLs or 5 whole spine FCLs in T1-weighted MRI would suggest a diagnosis of axSpA with specificity comparable to that of the ASAS criteria. This could assist diagnosis while avoiding the costs of additional MRI of the SI joints.

A main problem with validation studies in axSpA is a lack of “gold standard” as there is no single investigation to define the disease. Similar to other international cohorts, we used diagnoses made by clinic rheumatologists as a gold standard. All studied patients had a clinical diagnosis before recruitment to minimise classification bias.

One of the limitations in our study was the small sample size in the control group. Another limitation was that the effect of age and disease duration were not taken into account during the evaluation of FCLs. According to Weber et al. and the SPACE cohort [7], aging may increase the number of FCLs [6]. However, patients in our study cohort were relatively young, and FCLs were uncommonly found in the non-axSpA group. Therefore, the effect of aging had minimal contributions to the prevalence of FCLs in our study. Since patients in tertiary care settings inherently have increased pre-test probability, the use of FCLs in patients with chronic back pain in primary care or in the general population might have different implications.

## Conclusion

FCLs could be used to diagnose axSpA but routine use is not recommended. Notwithstanding this, in patients with clinical features of axSpA and the presence of at least 3 anterior thoracic FCLs or 5 whole spine FCLs in T1-weighted MRI spine suggests a diagnosis of axSpA without additional MRI of the SI joints.

## Additional file

Additional file 1: Using fatty corner lesions for diagnosis of axial spondyloarthritis data file. (SAV 55 kb)
